# Preeclampsia and Future Cardiovascular Risk: Are Women and General Practitioners Aware of This Relationship? The Experience from a Portuguese Centre

**DOI:** 10.1155/2014/531539

**Published:** 2014-05-28

**Authors:** Pedro Viana Pinto, Mariana Rei, Ana Paula Machado, Nuno Montenegro

**Affiliations:** ^1^Serviço de Ginecologia-Obstetrícia, Centro Hospitalar São João, Alameda Professor Hernâni Monteiro, 4200-319 Porto, Portugal; ^2^Faculdade de Medicina da Universidade do Porto, Alameda Professor Hernâni Monteiro, 4200-319 Porto, Portugal

## Abstract

*Objective*. To evaluate the impact of preeclampsia in the modification of lifestyle habits and decreasing cardiovascular risk factors in a population of women at least 6 months after having the diagnosis of preeclampsia. *Methods*. Cross-sectional observational study. Data included 141 cases of preeclampsia and chronic hypertension with superimposed preeclampsia on singleton births diagnosed in our institution between January 2010 and December 2013. From the cases diagnosed over 6 months a standardized questionnaire evaluating lifestyle changes was applied. *Results*. We reviewed 141 cases, of which 120 were diagnosed for more than 6 months. An overall participation rate in the questionnaire of 65% was yielded. A slight increase from the mean BMI before pregnancy was found. No statistical significant association was established between postpregnancy mean BMI, weight variation, and the frequency of aerobic exercise with the severity of preeclampsia. Only 28% of our cases were practising aerobic exercise at least weekly. The majority of women assessed blood pressure at least monthly (45/78), but only 25 assessed glycaemia at least once/year. *Conclusion*. This study shows that the majority of our patients and general practitioners do not take into consideration a previous pregnancy affected by preeclampsia as a risk factor for future cardiovascular disease.

## 1. Introduction

The hypertensive disorders of pregnancy remain one of the most important causes of maternal and fetal morbidity and mortality all over the world. Preeclampsia, either alone or superimposed on preexisting hypertension, affects around 5 to 8% of all pregnancies and is responsible for approximately 50,000 maternal deaths annually [1]. Although appropriate perinatal care has reduced the number and extent of poor outcomes, serious maternal and fetal morbidity and mortality still occur [2].

Preeclampsia is now clearly associated with an anomaly of the placentation and incomplete remodelling of the uteroplacental spiral arteries [3]. Trophoblast invasion is often defective in preeclampsia, particularly in early-onset preeclampsia, affecting the endovascular, but not the interstitial, invasion pathway. The resulting abnormal uteroplacental flow is associated with placental oxidative and endoplasmic reticulum stress, probably from ischemia-reperfusion injury, which stimulates the release of proinflammatory cytokines and imposes an excessive inflammatory stress on the maternal circulation [4, 5]. This systemic inflammation may result from a variety of circulating factors such as pro- and antiangiogenic proteins (sFLT1, placental growth factor or soluble endoglin), proinflammatory cytokines, and activating autoantibodies against the AT1-receptor [4, 6–8]. Recently, transcription factors (like HIF-1*α* and NF-kappaB) and cell stress induced genes like GADD45*α* have been established as linking points between the hypoxic aggression and the release of antiangiogenic factors [9, 10]. The key target of these factors is the maternal vascular endothelium, which plays an important role in smooth muscle tone control and regulation of the coagulation and fibrinolytic systems. Alterations in the concentration of circulating markers of endothelial dysfunction have been consistently reported in women with preeclampsia, highlighting the role of the endothelium in the pathogenesis of this disease [11, 12].

Acute atherosis is a vascular lesion observed in approximately 20 to 40% of the maternal spiral arteries. These lesions, mainly affecting the downstream of the unremodelled spiral arteries, strongly resemble atherosclerosis and have also been implied in the pathogenesis of preeclampsia [4, 8]. An immune mediated reaction may be responsible for the development of these lesions with maternal immune recognition of foreign fetal HLA-C leading to a switch from the anti-inflammatory milieu of normal pregnancy to a more proinflammatory status, triggering the formation of lipid-filled foam cells and vascular fibrinoid necrosis within the uterine arteries vascular wall [4, 13–15]. These findings enhance the idea that alloreactivity between maternal decidual immune cells and fetal extravillous trophoblasts may also contribute to the pathogenesis of preeclampsia [14, 15].

Several studies have clearly demonstrated that women with a history of preeclampsia have an increased risk of 2–4-fold cardiovascular diseases (CVD) later in life, at least equalling the risk attributed to obesity and smoking [16–19]. This situation is important to such an extent that led the American Heart Association, in 2011, to consider preeclampsia as a major risk factor for cardiovascular diseases, mainly hypertension, myocardial infarction, stroke, and diabetes [2, 20]. The probability seems to be higher if it is recurrent or early-onset preeclampsia or when it is associated with preterm birth and fetal growth restriction [18, 19, 21]. Possible explanations for this cardiovascular profile include the following: (1) both cardiovascular disease and preeclampsia share risk factors including dyslipidemia, increased insulin resistance, hypertension, obesity, and endothelial dysfunction, turning pregnancy into a “stress test” with the development of hypertensive disorders during pregnancy identifying a woman destined to develop cardiovascular disease; (2) pregnancy, and especially preeclampsia, may induce permanent arterial changes—the proatherogenic stress of pregnancy, excessive in many women with preeclampsia, could activate arterial wall inflammation that fails to resolve after delivery, increasing the risk for future cardiovascular disease [4, 19].

Therefore, women with a history of preeclampsia should be encouraged to have a more rigorous follow-up and adopt a healthier lifestyle. Patient and healthcare provider education is essential for the successful assessment and management of cardiovascular risk and prevention of the long term burden associated with preeclampsia.

The aim of this study was to evaluate the impact of the diagnosis of preeclampsia in the modification of lifestyle habits and decreasing cardiovascular risk factors in a population of women at least 6 months after having the diagnosis of preeclampsia, in a tertiary hospital for a 4-year period. Furthermore, we intended to evaluate the patient and general practitioner knowledge of the cardiovascular risks associated with a history of preeclampsia.

## 2. Methods

The 141 cases of preeclampsia and chronic hypertension with superimposed preeclampsia with singleton births diagnosed in our institution from January 2010 to December 2013 were retrospectively reviewed. Patients with multiple pregnancy were excluded.

We defined preeclampsia as the new onset of hypertension and either proteinuria or end-organ dysfunction after 20 weeks of gestation in a previously normotensive woman. Superimposed preeclampsia was defined as preeclampsia complicating chronic hypertension, according to The American College of Obstetricians and Gynecologists (ACOG) criteria. Systolic blood pressure of 160 mmHg or higher or diastolic blood pressure of 110 mmHg or higher in two occasions, thrombocytopenia (less than 100,000/microliter), impaired liver function (elevated blood concentrations of liver enzymes to twice normal), or severe refractory epigastric pain, progressive renal insufficiency (serum concentration greater than 1.1 mg/dL or a doubling concentration), pulmonary edema, or new onset cerebral/visual disturbances were considered severe features of preeclampsia. The Mississippi classification was used to define HELLP syndrome: hemolysis (increased LDH level and progressive anemia), hepatic dysfunction (LDH level > 600 IU/L, AST > 40 IU/L, ALT > 40 IU/L or both), and thrombocytopenia (platelet nadir less than 150,000 cells/mm^3^). The term fetal growth restriction was used to describe fetuses with an estimated weight less than the 10th percentile for gestational age.

Demographic and outcome data were collected from the computerised hospital database, VCIntegrator Obscare, which records all the final diagnoses by patient, and a systematic search using preeclampsia, superimposed preeclampsia, and HELLP syndrome as keywords was carried out. Demographic variables collected included woman's age at delivery, prepregnancy maternal body mass index (BMI), parity, education, past obstetric history (including previous preeclampsia, gestational diabetes, preterm birth, and fetal death), the indication for labour induction, and the mode of delivery (vaginal birth or caesarean section). Pregnancy adverse outcomes as fetal growth restriction, gestational diabetes, preterm birth, abruption placentae, and HELLP syndrome were also collected.

A standardized telephonic questionnaire was applied to 120 women who were diagnosed with preeclampsia or superimposed preeclampsia between January 2010 and October 2013. We excluded the 21 cases that delivered in the last six months, to better evaluate women lifestyle after hospital's follow-up and maternal license conclusion. Forty-two women not answering after four telephonic calls in three consecutive days were considered nonresponders. For those who were contacted successfully, the purpose of the study was explained and an invitation was given to participate in the evaluation.

The questions included current age, weight, medication (including antihypertensive drugs, insulin or oral antidiabetic drugs, antidyslipidemic drugs, aspirin, or others), and contraception method (none, barrier, combined hormonal, progestin-only pills, subdermal implant, vaginal ring, intrauterine device, and female sterilization). We inquired about the mean meals per day, healthy nutrition behaviour, and attendance to nutritional counselling and to general medicine consultation and if an explanation of preeclampsia subject was addressed. Aerobic exercise practice before and after pregnancy was graded as (i) none, (ii) once per month, (iii) once per week, (iv) twice weekly, and (v) more than twice weekly. The monitoring of tensional values and glycemic levels were graded as (i) never, (ii) once per year, (iii) once per month, and (iv) once per week.

Study data were collected, validated, and entered into a dedicated study database by trained personnel. A descriptive analysis was performed using SPSS 20.0 software and the STATISTICA FOR WINDOWS statistical package, version 10.0. Chi-square test was used to compare categorical variables and one-way ANOVA test was applied to compare the means BMI between women with preeclampsia with or without severe features. No adjustment for confounders was made. A *P* value of less than 0.05 was considered significant.

## 3. Results

From January 2010 to December 2013 our institution admitted 141 cases of preeclampsia (22 cases of chronic hypertension with superimposed preeclampsia), with 120 cases having more than 6 months after delivery. A total of 78 women with previous preeclampsia answered the questionnaire, resulting in an overall participation rate of 65%. The remaining women did not answer the questionnaire.

Concerning the demographic characteristics (Table 1), the median (range) maternal age was 31 years (15–46). The mean BMI before pregnancy was 27.73. Seventy-five women were nulliparous (53.2%) and, from the multiparous women, 23 had a previous preeclamptic pregnancy. Along with preeclampsia, 37 women had suspected intrauterine growth restriction (all of them gave birth to small for gestational age newborns) and 64 newborns were preterm; the mean weight of newborns was 2533.77 g (400–4170 g). During the study period 47 women suffered from severe preeclampsia (29 before 34 weeks of gestation) and 16 from HELLP syndrome. Labour induction was performed in 93 deliveries (66%), from which 37 (39%) ended up in a caesarean section, nonreassuring fetal tracing (14 cases), failed induction of labour and arrested labour (both with 8 cases) being the most common reasons. The overall caesarean section rate was 52%.

Regarding the questionnaire (Table 2), the mean time from preeclampsia diagnosis was 2.02 years. Most women were taking combined hormonal pills as their contraceptive method. Eight women were trying to conceive again and all of them replied not knowing that they required a more rigorous follow-up during their next pregnancy.

**Figure 1:**
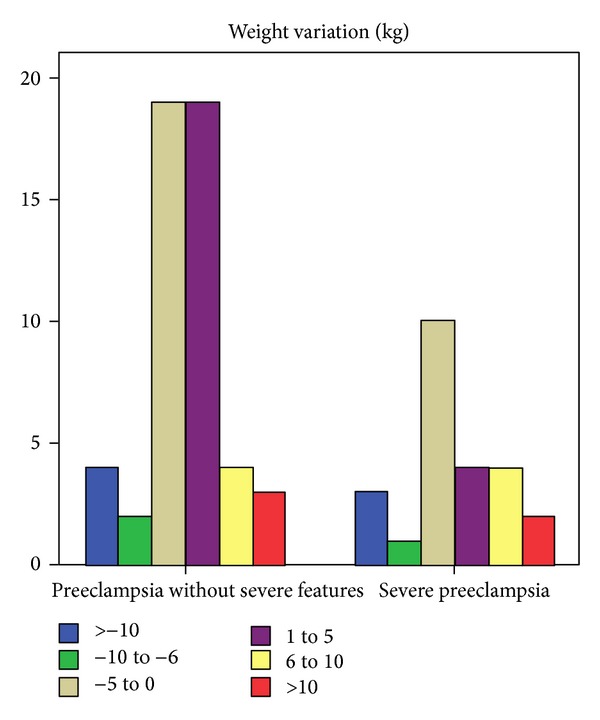
Weight variation distribution in women with preeclampsia without severe features and with severe preeclampsia.

The mean BMI was 26.7, demonstrating a slight increase from the mean BMI before being pregnant (26.07). Notably, 29 women diminished their weight while 35 increased weight (mean increase of 6,1 Kg). We could not find any statistical significant difference (Table 3) in postpregnancy mean BMI between women with severe preeclampsia and those with preeclampsia without severe features. Moreover, a statistical significant association could not be established between the severity of preeclampsia and the weight variation from pre- to postpregnancy (Figure 1). However, when comparing women with preeclampsia with women with chronic hypertension with superimposed preeclampsia there was a statistically significant reduction (*P* = 0.006) in weight in women with superimposed preeclampsia. The mean number of meals per day per patient was 4.74, ranging from 2 (1 woman) to 8 (3 women). Thirty-four women claimed a healthy change in diet, mostly salt intake reduction (considering less relevant the need for other adjustments) by their own initiative (only 11 had nutritional counselling).

Regarding aerobic exercise, only 6 women reported having increased the frequency of aerobic exercise practice comparing to before pregnancy, while 45 did not alter the frequency of exercise after having a pregnancy complicated by preeclampsia. Consistently, women affirmed that they knew about the importance of regular exercise practice but they did not change their habits. Only 28% of our cases were doing aerobic exercise at least weekly, with 9 women reporting exercise more than twice a week (whereas 21 were engaged in exercise with this frequency before the case index). Fifty-four women stated not doing any exercise at all. Regarding the frequency of aerobic exercise practice, no significant statistical difference was found between women with severe preeclampsia and those without severe features.

Lastly, women reported having a mean of 2.44 appointments per year with their basic healthcare provider, ranging from 0 (8 women) to 10 (5 women), with the majority (33) having one appointment per year. Furthermore, 24 women stated that their basic healthcare provider had addressed the item preeclampsia and future cardiovascular risks implied. The majority of women feared the possibility of developing chronic hypertension, with 45 women assessing their blood pressure at least every month (10 women remained hypertensive after pregnancy).

Most women did not find it important to assess the fasting blood glucose, with 53 women not doing it once a year. Not surprisingly, women with previous gestational diabetes were more sensitized to this issue, with 71% assessing fasting blood glucose at least once a year against 28% with preeclampsia and without gestational diabetes.

## 4. Discussion 

Cardiovascular disease (CVD) is the leading cause of death in women in all developed countries [19]. Despite being more frequent in male, the disease in women is now looked at differently all over the world. In 2007, CVD caused one death per minute among women in the United States [22]. In Portugal, during 2010, the mortality rate attributable to CVD in women was 342,7/100 000, making it the leading cause of death in this gender [23]. Pregnancy is being regarded as a cardiovascular risk “stress test” and so more emphasis is being paid to past obstetric history. It is now quite established that a hypertensive disorder occurring during pregnancy, particularly preeclampsia, identifies a subset of women with increased risk of developing CVD. A recent large meta-analysis found that women with a history of preeclampsia have an increased risk for subsequent ischemic heart disease, stroke, and venous thromboembolic events over 5 to 15 years after pregnancy [24]. Risk factors for preeclampsia, resembling those for atherosclerosis, are increasing in prevalence, stressing its importance as a future CVD predictor. This is highlighted in the 2011 update of the American Heart Association “effectiveness-based guidelines for the prevention of cardiovascular disease in women,” with preeclampsia being classified as a major risk factor for future CVD [20]. The European Society of Cardiology also states the importance of a pregnancy complicated by preeclampsia as a risk factor, recommending annual vigilance of blood pressure and metabolic factors as well as lifestyle modifications. The Portuguese Society of Cardiology has introduced these recommendations in practice guidelines in 2011 [25, 26]. The American College of Obstetricians and Gynecologists recommends a yearly assessment of blood pressure, lipids, fasting blood glucose, and body mass index after having a preeclampsia [2].

An aim of this study was to highlight the value of lifestyle modifications and to encourage clinicians to consider cardiovascular risk assessment in women with a previous preeclampsia. Awareness of a history of preeclampsia might allow the identification of cases not previously recognized as at-risk for CVD, allowing the implementation of measures to prevent the occurrence of these events. Our population did not appear sensitized to adopt a healthier lifestyle, as shown by a slight increase on their mean BMI with a decrease in the practice of aerobic exercise. Also, their diet was similar as before pregnancy, and when a change on the diet was reported, the only modification was a reduction in salt intake without any professional counselling. Besides the reduction in salt intake, based on the dietary approaches to stop hypertension (DASH diet) introduced in 1998, the ACOG advocates a diet rich in fibers, vegetables, and fruits and low in fat [2, 27]. We believe that professional counselling may improve the adhesion to a healthier diet, reducing patient's cardiovascular risk.

The majority of our patients are not engaged in regular exercise, with a great number of them stating a decrease in the practice of regular physical exercise. From the 21 women engaged in regular aerobic exercise (more than twice per week) before pregnancy only 6 remained practicing exercise with the same regularity after having a preeclampsia. This probably reflects a trend towards a sedentary lifestyle in the Portuguese women. The implementation of strategies to increase the time spent doing exercise seems crucial to improve health and quality of life of these women, reducing the burden imposed by this disease.

In Portugal, regular medical follow-up is provided by general practitioners, including the great majority of women during and after pregnancy. From our study we can assume that the majority of general practitioners (70%) do not take into consideration the preeclampsia issue, probably meaning that they are not aware of the future implications of preeclampsia in CVD, and are not taking into consideration previous obstetric history when assessing cardiovascular risk profiles. Furthermore, the association between preeclampsia and future development of diabetes is seriously undervalued, both by general practitioners and by patients, as shown by the number of women not making any fasting blood glucose assessment after pregnancy. On the other hand, women are more conscious of the future risk of developing hypertension, with 17 women assessing blood pressure once a week. This probably reflects an association established on an individual basis, reflecting the more straightforward connection between preeclampsia and hypertension.

Much has to be done in order to improve the follow-up of these patients. As a beginning step, in our institution we implemented a specialized postpartum appointment for all women with a pregnancy complicated by a hypertensive disorder with the aim of explaining the risks for a future pregnancy, the lifestyle modifications, and the surveillance that should be implemented in order to reduce the risk of future CVD.

To our knowledge there is not much information in our country regarding the follow-up of women with a history of a pregnancy complicated by preeclampsia. Although the results were basically what we expected, our study proves that much has to be done in order to guarantee the ideal monitoring of these patients. We found satisfactory the overall participation rate of 65%, chiefly because every woman answering the phone call accepted to participate in our study.

Some limitations of the study should be addressed. Above all, the small number of years of follow-up (mean time from preeclampsia diagnosis was 2.02 years) prevents us from evaluating long term outcomes for these women. The implications for the future cardiovascular profile are long term rather than short term, precluding us from taking strong inferences regarding future risk for these patients. However, lifestyle modifications and adequate follow-up by general practitioners should be initiated right away in order to prevent CVD, supporting our conclusions. Also, although prepregnancy weight and height were obtained from birth certificates with reliable information, after pregnancy weight was obtained by a telephone call, this information may have suffered from some bias, as estimates of obesity prevalence based on self-reported weight tend to be lower than those based on measured weight. Therefore, if we underestimated the rate of obesity, we have also underestimated our conclusions; the same goes for aerobic exercise. Another limitation of our study regarding its retrospective nature is that we might have missed some diagnosis, especially due to coding errors, but, most likely, no cases of severe preeclampsia, with a major impact on future CVD, were missed.

## 5. Conclusion 

CVD is the leading cause of women mortality. Considering preeclampsia as a risk factor for CVD it is of uppermost importance to take it into consideration when assessing for cardiovascular risk in women. It is also important for these women to adopt a healthier lifestyle, including a balanced diet, more regular exercise, and losing weight as mainstays for preventing future cardiovascular events. With this study we demonstrate that most of our patients previously affected by preeclampsia are not aware of the risks for their future life. Although most of them are aware of the probability of developing hypertension, they are not inclined to change their lifestyle. As a consequence, the majority of our cases have failed to change their routines and in some cases they have even implemented a worse lifestyle. It is imperative to pay more attention to these women in order to provide the best assessment according to the currently considered ideal standards. This study aims to highlight the value of lifestyle modifications and to encourage clinicians to consider cardiovascular risk assessment and active management in women with a previous preeclampsia.

## Figures and Tables

**Table 1:** Participant baseline characteristics during pregnancy.

Participant baseline characteristics during pregnancy	
Age (mean)	30.82
Body mass index before pregnancy (mean)	27.73
Education (%)	
Primary school	16 (11.3)
Secondary school	63 (44.6)
University	61 (43.6)
Conception (%)	
Spontaneous	135 (95.7)
Medically assisted	6 (4.3)
Parity (%)	
Primiparous	75 (53.2)
Multiparous	66 (46.8)
Past obstetric history	
Previous preeclampsia	23
Gestational diabetes	3
Fetal death	4
Preterm birth	12
Pregnancy adverse outcomes	
Fetal growth restriction	37
Severe preeclampsia	47
Gestational diabetes	16
HELLP syndrome	16
Placenta abruption	3
Preterm birth	64
Gestational age at birth (mean)	35.82
Type of labor	
Induced	93
Obstetric cholestasis	1
Fetal death	4
Preeclampsia	72
Severe preeclampsia	15
HELLP syndrome	1
Spontaneous	48
Mode of delivery	
Vaginal birth	68
Caesarean section	73
Severe preeclampsia/HELLP syndrome	20
Nonreassuring fetal tracing	18
Failed induction of labor	8
Arrested labor	12
Fetal malpresentation	7
Previous caesarean section	6
Fetal anomaly	2

**Table 2:** Baseline characteristics of women answering the questionnaire.

Baseline characteristics of women answering the questionnaire	
Age (mean)	25.46
Current body mass index (mean)	26.7
Contraception	
None	8
Barrier	10
Combined hormonal	29
Progestin-only pills	9
Subdermal implant	7
Vaginal ring	1
Intrauterine device	10
Female sterilization	4
Mean meals per day	4.7
Aerobic exercise before pregnancy	
None	41
Once per month	3
Once per week	7
Twice weekly	6
>twice weekly	21
Aerobic exercise after pregnancy	
None	54
Once per month	2
Once per week	6
Twice weekly	7
>twice weekly	9
Appointments with healthcare provider per year (mean)	2.44
Approach to preeclampsia by healthcare provider	
Yes	24
No	54
Blood pressure assessment	
Never	7
Once per year	26
Once per month	28
Once per week	17
Glycemia assessment	
Never	53
Once per year	19
Once per month	5
Once per week	1
Chronic hypertension after pregancy	10

**Table 3:** Association between preeclampsia severity and lifestyle modifications.

	Severe preeclampsia		
	Yes	No	
BMI postpregnancy	25.2	26.4	*P* = 0.323
Aerobic exercise after pregnancy			*P* = 0.837
(i) Increased	3	3	
(ii) Decreased	9	18	
(iii) Did not change	14	31	
Weight variation-Kg (%)			*P* = 0.541
(i) >−10	3 (12.5)	4 (7.8)	
(ii) −10 to −6	1 (4.2)	2 (3.9)	
(iii) −5 to 0	10 (41.7)	19 (37.3)	
(iv) 1 to 5	4 (16.7)	19 (37.3)	
(v) 6 to 10	4 (16.7)	4 (7.8)	
(vi) >10	2 (8.3)	3 (5.9)	
